# Sustain-release lipid-liquid crystal formulations of pexiganan against *Helicobacter pylori* infection: in vitro evaluation in C57BL/6 mice

**DOI:** 10.1186/s40360-024-00731-z

**Published:** 2024-01-11

**Authors:** Kiarash Ghazvini, Hossein Kamali, Hadi Farsiani, Masoud Yousefi, Masoud Keikha

**Affiliations:** 1 Department of Microbiology and Virology, Mashhad University of Medical Sciences, Mashhad, Iran; 2 School of Pharmacy, Mashhad University of Medical Sciences, Mashhad, Iran; 3 Department of Microbiology, School of Medicine, Iranshahr University of Medical Sciences, Iranshahr, Iran

**Keywords:** *Helicobacter pylori*, C57BL/6, Infection, Cure rate: liquid crystal

## Abstract

**Introduction:**

The Gram-negative bacterium *Helicobacter pylori*, *H. pylori*, is associated with significant digestive disorders. However, the effectiveness of bacterial eradication is declining due to drug resistance. A potent anti-*H. pylori* activity is shown by the natural antimicrobial peptide pexiganan.

**Objective:**

The current study aimed to evaluate the effectiveness of pexiganan and its lipid-liquid crystals (LLCs) in inducing *Helicobacter pylori* in mice.

**Methods:**

In this experimental study, *H. pylori* infection was first induced in C57BL/6 mice. Secondly, the antibacterial efficacy of pexiganan and its LLCs formulations was investigated to eliminate *H. pylori* infection.

**Results:**

The *H. pylori* infection could not be completely eradicated by pexiganan peptide alone. However, incorporating pexiganan within the LLC formulation resulted in an increased elimination of *H. pylori*. Under the H&E strain, the pexiganan-LLCs formulation revealed minimal mucosal alterations and a lower amount of inflammatory cell infiltration in the stomach compared to the placebo.

**Conclusion:**

Clarithromycin was more effective than pexiganan at all tested concentrations. Furthermore, the pexiganan-loaded LLCs exhibited superior efficacy in curing *H. pylori* infection in a mouse model compared to pexiganan alone. This formulation can enhance *H*. *pylori* clearance while mitigating the adverse effects, typically associated with conventional drugs, leading to a viable alternative to current treatment options.

## Introduction

A gram-negative, flagellated, microaerophilic bacterium known as *Helicobacter pylori* (*H. pylori*) was found in the stomach mucosa of about 4 billion individuals worldwide [1]. A *H. pylori* infection is linked to serious digestive disorders, such as gastric cancer, chronic gastritis, peptic ulcers, and gastric atrophy [2]. Therefore, treating conditions like peptic ulcer disease and mucosa-associated lymphoid tissue (MALT) lymphoma primarily aims to eradicate *H. pylori* infection. When the *H. pylori* infection is eradicated in patients, who undergo endoscopic resection, there is a notable decrease in their risk of developing metachronous gastric cancer [3]. Additionally, not only is *H. pylori* eradication not always successful, but the high cost of treating *H. pylori* infections is also considered one of the major obstacles to therapy, along with other challenges such as side effects and low patient compliance [4]. The phenomenon of antibiotic resistance, particularly the advent of multi-drug-resistant (MDR) *H. pylori* as the primary concern, has recently decreased the cure rate to below 80% worldwide [5]. Due to antibiotic resistance, *H. pylori* has been ranked by the World Health Organization (WHO) as one of the top 20 pathogens and the greatest hazard to human health since 2017 [6].

A class of small peptides with broad-range antibacterial action, either synthetic or natural in origin, is known as Antimicrobial Peptides (AMPs) [7]. Based on the recent data, traditional mechanisms of resistance often have little effect on AMPs (7–8). Pexiganan is a naturally occurring 22-amino acid peptide extracted from the skin of African clawed frogs, showing antimicrobial efficacy against 3109 clinical isolates of gram-positive, gram-negative, and anaerobic bacteria [8]. Under the MSI-78 brand, pexiganan is being investigated in a third round of clinical trials to treat diabetes patients’ foot ulcers [9]. Pexiganan exhibits significant antibacterial activity against *H. pylori* infections, resulting in a promising option for the development of new therapeutic medicines targeted at effectively treating *H. pylori* infections [10]. Therefore, further research is needed to gather more evidence and determine the true potential of pexiganan as a treatment option for *H. pylori* infections.

On the other hand, *H. pylori* remains in the submucosal area, and the antibiotic is largely trapped in the gastric mucosa. Acidic conditions in the stomach environment result in the degradation and reduction of the residence time of antibiotics in the stomach [11]. Accordingly, mucoadhesive delivery of antibiotics leads to protection from antibiotics within the acidic pH, increased residence time in the stomach and local drug concentration, and the closing of mutation windows [12]. Hydrophobic peptide-based therapeutics, such as AMPs, have garnered significant interest among researchers for the treatment of resistant infections in recent years. However, several limitations, such as low solubility, poor metabolic stability, inadequate oral bioavailability, and rapid elimination, render these biomolecules unsuitable for clinical use [13]. Liquid Crystal Nanoparticles (LCNs) present a viable solution to these concerns, as they are easy to use and affordable with a quick production process. The LCNs are composed of biocompatible and non-toxic excipients rather than polymer nanoparticles, enhancing the potential for oral delivery of LCNPs. Additionally, LCNs can be incorporated with biomolecules to protect them from the effects of harsh gastrointestinal conditions [14]. LCNs have the capability to significantly reduce drug toxicities due to their sustained drug-release properties [15]. LCNs in both thermotropic and lyotropic crystal forms are known; lyotropic crystals are more commonly employed in the pharmaceutical industry. The preparation of lyotropic crystals involves combining a mixture of amphiphilic molecules with a hydrophobic solvent at a specific temperature [16].

The lyotropic structure is classified into three forms, including bicontinuous cubic (Q_2_), lamellar (Lɑ), and reverse hexagonal (H_II_). Drugs are routinely released from the H_II_ phase more slowly than the Q_2_ phase [17]. Glycerol dioleate (GDO), Soybean Phosphatidylcholine (SPC), and N-methyl-2-pyrrolidone (NMP) are the most important basic components of the liquid crystal-forming gels. The NMP spreads slowly to the vicinity, and water from the environment penetrates into the LCN structure. The structure of LCNs undergoes phase inversion, and the incorporated drug is slowly released [18]. No study has evaluated the effect of pexiganan delivery with LCNs on *H. pylori* eradication. The present study aimed to use lipid-liquid crystals for oral delivery of pexiganan for the elimination of *H. pylori* infection in C57BL/6 mice. The LCN phase dispersion formulation has several advantages, as follows: (1) protecting the drug from degradation in the Gastrointestinal (GI) tract; (2) lyotropic characteristics, which have a hydrophilic external surface and easily connect to the cells of the endothelial layer of the GI tract; (3) easily penetrating cubic phase across the endothelial cell membrane and incorporating pexiganan, with a hydrophilic structure, into itself and preventing the initial burst release phenomenon [19].

## Materials and methods

### Pexiganan synthesis and purification

Recombinant pexiganan was cloned, expressed, and purified in accordance with earlier instructions. *Pichia pastoris* GS115 was selected as a eukaryotic expression system to express the pexiganan antimicrobial peptide, which has the sequence NH_2_-GIGKFLKKAKAKFGKAFVKILKK-COOH, to accomplish this purpose (Accession number: MK515150). The final concentration of expressed pexiganan was measured at 54 µg/L after purification [20].

### Preparation of formulations

A total of 10 milligrams (mg) of pexiganan were dissolved in NMP to create a pexiganan solution. Additionally, a combination of 250 mg of soy phosphatidylcholine and 250 mg of glycerol dioleate was used to produce the LLC vehicle. To create the LLC-GDO formulation, the pexiganan solution was combined with the LLC vehicle and heated to 60 °C for one hour. The development of phase dispersion in the LLC-GDO formulation was evaluated using a polarized light microscope (PLM).

### In vitro release assay

The LLC formulations were introduced to vials using a 20-gauge needle filled with 75 ml of release media consisting of PBS, Tween 80 (2% w/w), pH 3.2 (achieved by adding HCl solution), and maintained at 37 °C. Upon injection into the medium, the vehicle formulation underwent a transformation into a gel-like phase. The vials were then placed in a shaking incubator at 37 °C and 100 RPM for the in vitro release study. The amount of released medication was measured in 2 milliliters of release media at predetermined time intervals of 2, 4, 6, 8, 12, 18, 24, 32, and 36 h. The removed sample was replaced with an equivalent volume to maintain the total volume of the media. Before HPLC analysis, the samples were centrifuged at 10,000 rpm for 15 min [21].

### Animals

Male C57BL/6 mice weighing between 18 and 25 gr and typically aged between 6 and 8 weeks were acquired from the Animal Center of Mashhad University of Medical Sciences. The animals were housed in polypropylene cages with standard circumstances authorized by the Animal Ethics Committee. These settings included free access to food and water, a temperature of 22 ± 2 °C, a humidity of 54%, and a 12-hour light/dark cycle. The Ministry of Health, Treatment, and Medical Education of Iran (2020) states that all animals were housed in compliance with national rules for animal care, and laboratory animals were utilized in scientific endeavors.

### Mice model of H. Pylori infection

An animal model of *H. pylori* infection was established using a clinical strain of *H. pylori*. The strain was obtained from a male patient with a peptic ulcer and was isolated in the clinical microbiology laboratory at Ghaem Hospital in Mashhad, Iran. In vitro evaluation using the E-test confirmed that the strain was susceptible to clarithromycin, metronidazole, amoxicillin, and tetracycline. Oral gavage with 0.3 mL BHI broth containing 10^9^ CFU of *H. pylori* was used to simulate an *H. pylori* infection (after fasting for three consecutive days with intervals). A total of 10 (*n = 6*) distinct groups were randomly selected from among all C57BL/6 mice. Pexiganan and pexiganan incorporated with Liposome-like Carriers (LLCs) were orally provided once a day for two weeks at a dosage of 1, 2, 5, or 10 mg/kg for 14 days after the inoculation. Accordingly, the mice in the control group had an *H. pylori* infection but did not receive pexiganan or antibiotics. When the treatment was finished, the mice were humanely euthanized with carbon dioxide (CO_2_) gas via compressed CO_2_ gas in cylinders in order to control the inflow of gas in a chamber. Gastric biopsies were collected and homogenized using a tissue homogenizer in 1.5 mL of BHI broth. The cure rate of *H. pylori* infection was determined by urease activity and microbiological culture under controlled microaerophilic conditions.

### Assessment of gastric mucoadhesion of LLC-pexiganan formulations

The mice were subjected to a 24-hour fasting period during the period of unrestricted access to water. Pexiganan and LLC formulations were orally administered at a final dose of approximately 10 mg/kg. After 4 h, the mice were humanely euthanized using CO_2_ inhalation. Gastric biopsies were collected, and the concentration of pexiganan was measured. The gastric biopsies were carefully rinsed three times with a PBS solution to ensure thorough washing. The washed solution samples were centrifuged at 2500× g for 5 min and at 8600× g for 10 min for pexiganan and its LLC formulations, respectively. The concentration of pexiganan was determined using the BCA Protein Assay Kit, as follows [22]:

$$\begin{gathered} Gastric\,mucoadhesion\,percentage\,as:\, \hfill \\ x = \frac{{the\,amount\,of\,pexiganan\,detected\,in\,supernatants}}{{the\,total\,amount\,of\,pexiganan\,added}} \times 100 \hfill \\ \end{gathered} $$

### In vivo toxicity assessment

There are two approaches for determining the toxicity of pexignan: acute toxicity evaluation after single-dose administration and sub-acute toxicity assessment after multiple-dose administration [23]. To estimate the in vivo LD_50_ value of pexiganan, a two-fold concentration gradient peptide injection was used in the acute toxicity experiment. Five different dosages (35 mg/kg, 17.5 mg/kg, 8.75 mg/kg, 4.37 mg/kg, and 2.18 mg/kg) were orally gavaged (p.o.) once and observed for five days in this manner. Furthermore, as previously mentioned [24], subacute toxicity was done to measure the cumulative harmful impact. In brief, the acute test determined the sub-lethal dosage of pexiganan, which was orally administered (p.o.) for five days. The animals were checked daily for mortality, changes in their hair, eyes, mucous membranes, and behavioral symptoms (salivation, tremors, convulsions, diarrhea, and lethargy). There is also biochemical examination of creatinine, urea, aspartate transaminase (AST), and alanine transaminase (ALT), hematological evaluation, and histological evaluation of blood samples and tissue biopsies, for example, liver, stomach, and kidney.

### Assessment of H. Pylori elimination

Stomach biopsy culture and urease assays were conducted to assess the extent of *H. pylori* infection. For the urease test, 0.1 ml of stomach homogenate was combined with a solution containing 3 ml of urea, including 0.25% phenol red, 1 mg/mL glucose, 1 mg/mL peptone, 5 mg/mL NaCl, and 2 mg/mL KH_2_PO_4_. The reaction was carried out at 37 °C for 4 h. Spectrophotometry was then used to measure the rate of urease enzyme activity at OD 550 nm. To ascertain if the *H. pylori* infection had been completely eradicated in the animals, the absorbance values at 550 nm and the standard deviations of the mean were compared to those of infected and untreated control mice [25]. The eradication of the *H. pylori* infection was also assessed using microbial culture. A total of 50 µl of stomach homogenate was inoculated onto Brucella agar medium supplemented with 10% horse blood and antibiotics (polymyxin B, amphotericin, and vancomycin) to cultivate the bacteria. The plates were incubated in a microaerophilic environment at 37 °C with 10% CO_2_ for 3–5 days. The growing colonies were then examined using colony histology, Gram staining, catalase, oxidase, and urease tests to confirm their identification as *H. pylori*. The number of bacterial colonies was counted on each plate to quantify the bacteria colonized in each mouse stomach, expressed as log CFU/g stomach [26].

### Histological evaluation

The antrum’s gastric biopsies were fixed using 10% formalin for tissue preservation. The fixed tissues were then embedded in paraffin to create paraffin-embedded (FFPE) blocks, sectioned into segments with a 5-millimeter microtome. Subsequently, the sections were stained with Hematoxylin-Eosin (H&E) to result in the visualization of cellular structures. Finally, based on established histological criteria, the stained stomach tissues were evaluated and graded for gastric inflammation using light microscopy.

### Statistical analysis

Non-parametrically distributed samples were analyzed using the Wilcoxon Mann-Whitney tests and one-way analysis of variance (ANOVA). The data was expressed as the mean ± standard error. Statistical significance was considered at a *p*-value less than 0.05 (*p*-value < 0.05). GraphPad Prism version 8 software was employed for the statistical analysis.

## Results

### Characteristics of LLC formulations

**Fig. 1:**
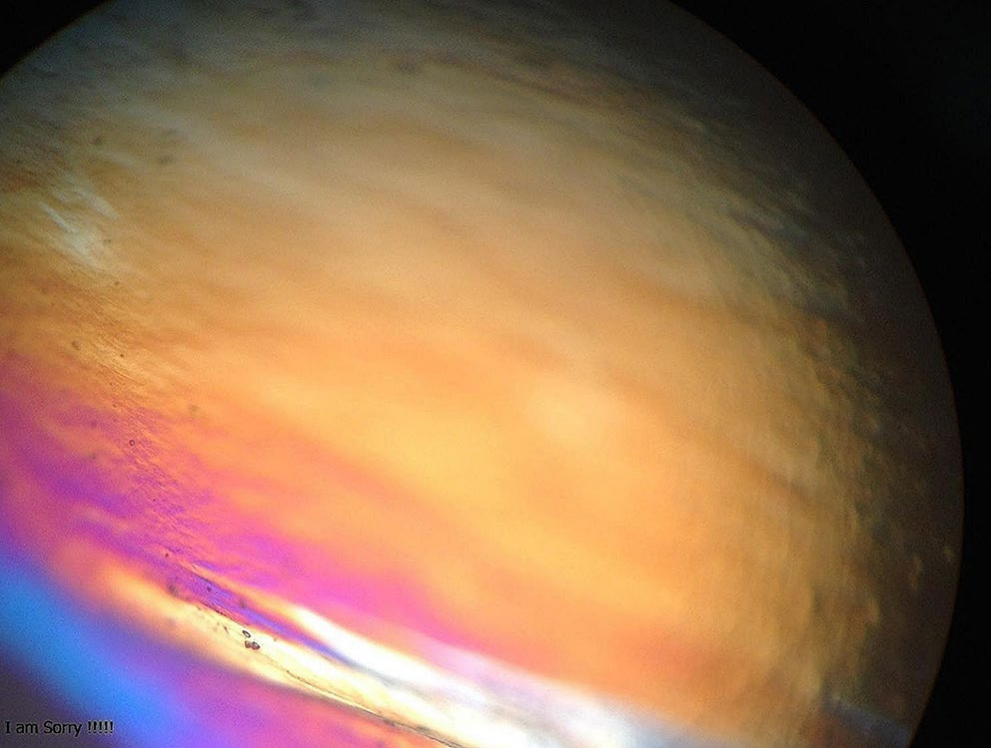
Images of GDO-based pexiganan-LLCs under a polarized light microscope

**Fig. 2:**
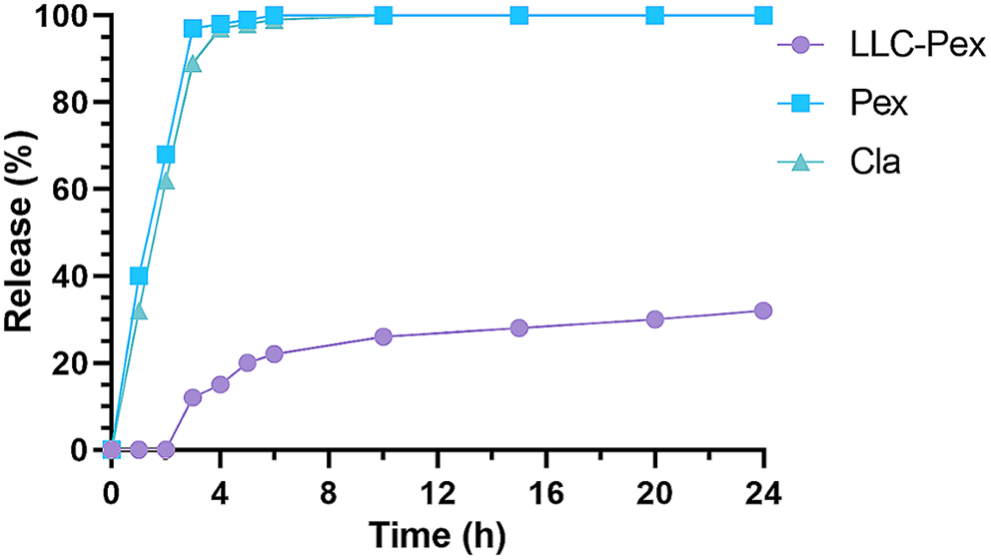
The cumulative release rate of pexiganan for LLC-GDO in-vitro. Cla: Clarithromycin; Pex: Pexiganan; LLC-Pex: Lipid Liquid Crystal-Pexiganan. Our results suggest that both clarithromycin as well as pexiganan have initial burst release without vehicle; However, pexiganan incorporated within lipid-liquid crystal formulation has slow-release than with no vehicles. As shown, pexiganan and clarithromycin does reach to more than 90% of release before the initial 4 h; While, the lipid liquid crystal has controlled the release of pexignan. In this relation, there are 20% of loaded pexignan was released from lipid liquid crystal formulation

The PLM was utilized to evaluate the phase change and liquid crystal texture structure of the LLC formulations. The anisotropic phases Lɑ and H_II_ were indicated by birefringence, which is a characteristic optical property. On the other hand, the cubic phase (Q_2_ phase) was identified by a lack of birefringence, resulting in a black backdrop in the PLM images [27]. The GDO-based formulations in combination with NMP, PC, and pexiganan at high water percentages exhibited a hexagonal (H_II_) phase structure, confirmed using PLM (Fig. 1). In contrast, the GDO vehicle formulations without NMP, PC, or pexiganan displayed a dark background when observed under polarized light microscopy (Fig. 1). The LLC-hexagonal (H_2_) phases contain more surfaces and more nano-channels. Thus, the H_2_ phases are proper candidates to create slow-release formulations [28]. The injection of H_2_ phases into a water solution can cause a transformation of the original phases, showing that the H_2_ phases may change into other phases. This phase of transformation can lead to the release of drug molecules from the system. Similarly, this phase transformation was observed after injecting LLC-pexiganan into an in vitro medium (PBS). The LLC-pexiganan formulation exhibited the H_2_ phase structure, which remained stable for at least 4 weeks under PLM. However, over a period of 35 days, water diffusion into the nano-structural system caused a change in the LLC phases, transitioning to cubic phases as observed under PLM. In summary, the release of pexiganan from LLC formulations is associated with the transformation of LLC phases. The release of pexiganan from LLC formulations exhibited a more uniform pattern compared to non-vehicle formulations (Fig. 2). Despite non-vehicle formulations, LLC formulations did not illustrate an initial burst release of pexiganan. In the in vitro study, the results of the observed drug release profiles were consistent with those of NMP release and degradation in vitro.

### In Vivo Bioadhesivity

**Fig. 3:**
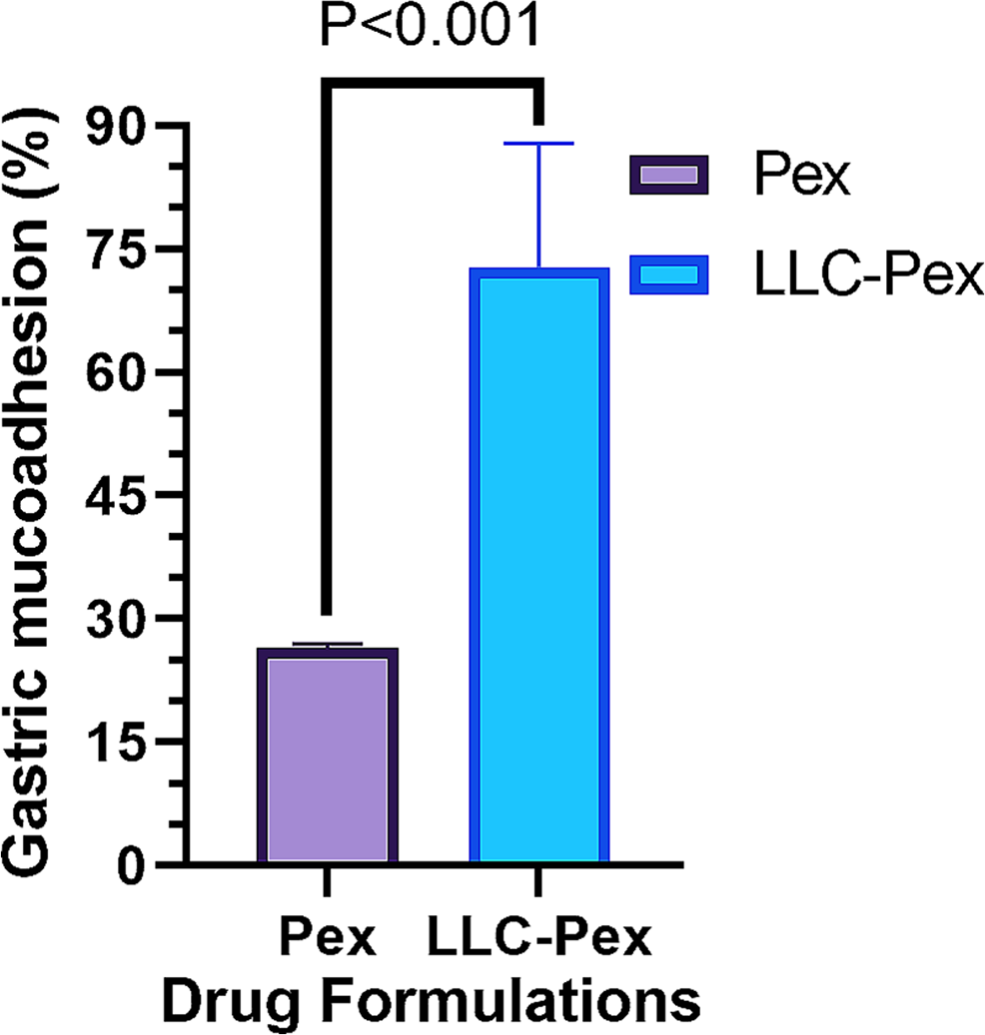
The evaluation of gastric bioadhesivity characteristics of pexiganan-LLCs

The gastric bioadhesivity of pexiganan was assessed in uninfected mice. After 4 h of oral administration, the remaining percentage of pexiganan was estimated at 26.5 ± 0.45%; however, the LLC formulation had a remaining percentage of 72.80 ± 15% (Fig. 3), showing that LLC formulations, enriched with GDO and PC can strongly bind to the mucous membranes of the stomach and Gastrointestinal (GI) tract. Therefore, these formulations with their high bioadhesive properties, can be considered promising options for oral drug delivery.

### In vitro toxicity results

**Fig. 4:**
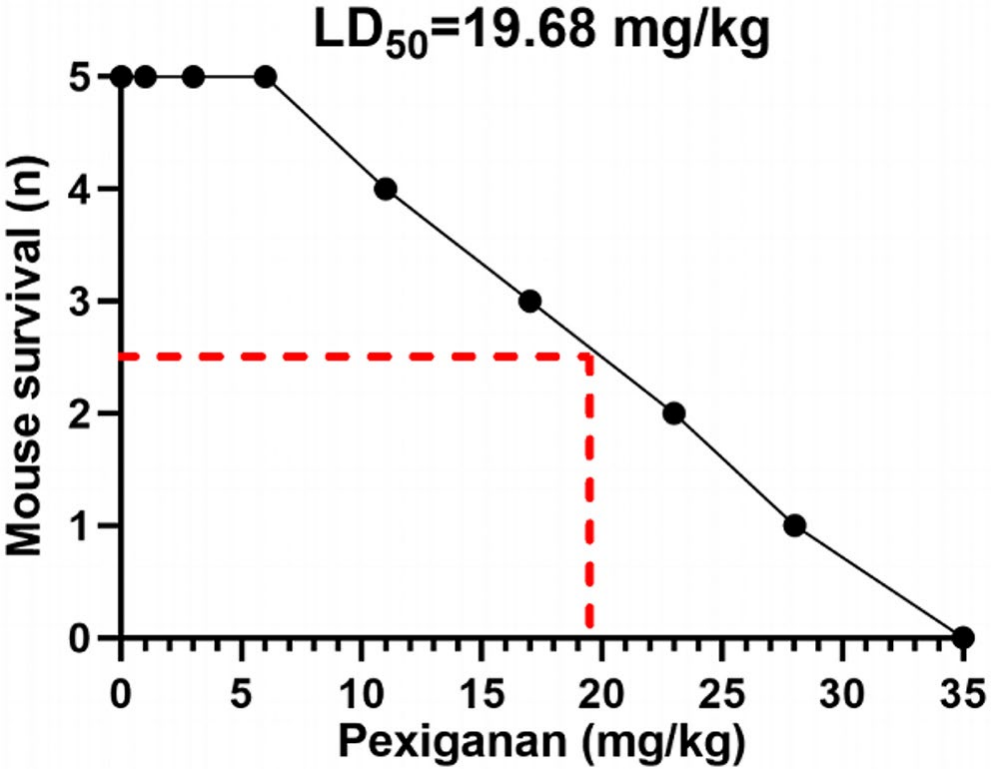
in vivo oral LD_50_ (median lethal dose) of pexiganan for C57BL/6 mice model

The results of our investigation showed that pexiganan’s oral LD_50_ value was 19.68 mg/kg. Pexiganan’s maximal sub-lethal dosage was 10.7 mg/kg (Fig. 4). During five consecutive days of continuous gavage of a sub-lethal dose of pexiganan in mice, cumulative toxicity measurements were performed. The results showed that there was no significant difference between the control group and the pexiganan-treated mice in terms of biochemical parameters, hematological features, or histopathological evaluations (Table 1).

**Table 1:** The impact of sub-lethal dose of pexiganan on biochemical and hematological parameters in mice

Group	Urea (mg/dL)	Creatinine (mg/dL)	AST (U/L)	ALT (U/L)	RBC (10^6^/µL)	WBC (10^3^/µL)
Control	47.39 ± 4.33	0.57 ± 3.25	77.63 ± 21.42	59.19 ± 21.37	9.27 ± 0.61	5.83 ± 0.02
Pexiganan (10.7 mg/kg)	45.25 ± 7.51	0.58 ± 2.37	75.02 ± 23.63	57.23 ± 13.35	9.15 ± 0.89	5.13 ± 0.52
*p*-value (t-test)	0.063	0.63	0.76	0.08	0.48	0.073

### In vivo H. pylori elimination rate

**Fig. 5:**
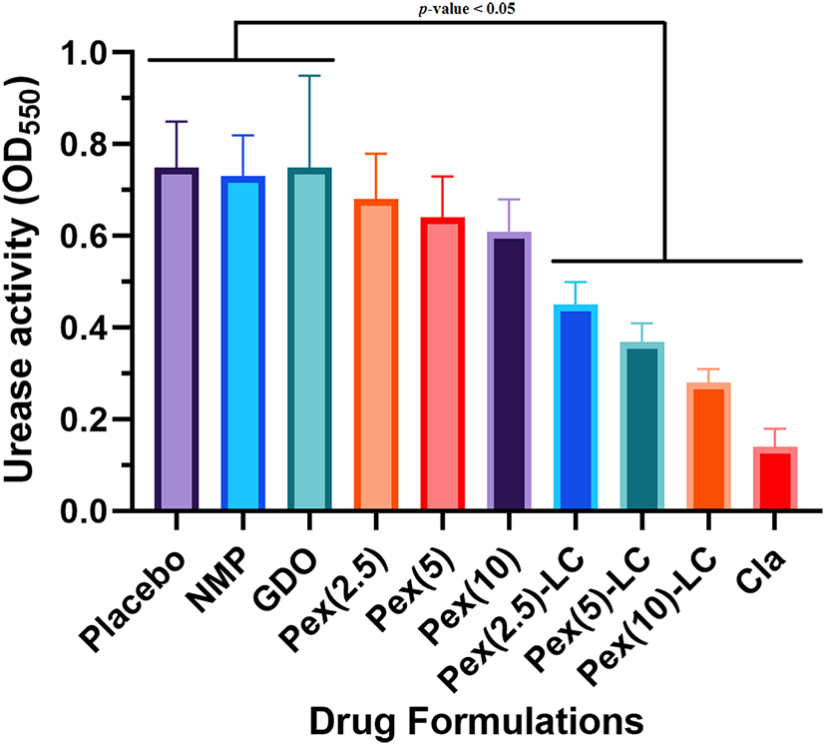
the assessment of clearance effectiveness of pexiganan against *H. pylori* infection based on urease activity tests

The oral LD_50_ dose of pexiganan-LLC formulation was determined in order to avoid toxicity or inflammatory cell infiltrations in the submucosa tissue as a result of the influence of pexiganan LLC components. To test the cure for *H. pylori* infection, the mouse models got the only sub-lethal dosage of pexiganan. The results of the urease test revealed a significant reduction in urease activities in the groups that received pexiganan and clarithromycin compared to those that received placebo (PBS buffer) (Fig. 5). Moreover, the group receiving pexiganan-containing lipid liquid crystals (Pex-LLCs) showed a significant difference in urease activity compared to the pexiganan intervention groups without a carrier (*p*-value < 0.05). However, no significant difference was observed between these groups in terms of clarithromycin. These results indicate that the clearance effect of Pex-LLCs is not as effective as that of clarithromycin.

Liquid lipid crystals strengthen the antimicrobial activity of pexiganan by decreasing peptide clearance from the blood and increasing delivery to the stomach. Table 2 presents the mean number of bacteria in the mice’s stomachs. The mean bacterial count in the mice of the control group without any drug delivery was measured at around 10^8^ CFU/stomach. However, the mean number of gastric bacteria in mice treated with pexiganan and oral clarithromycin decreased with increasing dose; complete clearance was not observed in any pexiganan-receiving group. In the current study, the cure rate was significantly different among the mouse groups. According to the results, the mice treated with clarithromycin had a significantly higher rate of *H. pylori* eradication compared to those treated with pexiganan and pexiganan-LLC formulations. Accordingly, clarithromycin is remarkably more effective in eliminating *H. pylori* infection compared to pexiganan and its lipid liquid crystal formulations. The superior effectiveness of clarithromycin in eradicating *H. pylori* infection highlights its incomparable efficacy in comparison to pexiganan and its associated lipid liquid crystal formulations.

**Table 2:** The in vivo assessment of *H. pylori* cure rates of pexiganan-conjugated lipid-liquid crystals

Drug formula	Dose (mg.kg)	Cure rate	Mean bacterial count (Log CFU/stomach)
Placebo	NA	0/6 (0%)	7.78 ± 0.61
NMP	NA	0/6 (0%)	7.18 ± 0.37
GDO	NA	0/6 (0%)	7.23 ± 0.56
Pexiganan	2	0/6 (0%)	6.68 ± 0.12
Pexiganan	5	0/6 (0%)	6.28 ± 0.35
Pexiganan	10	2/6 (33.3%)	4.26 ± 0.18
Clarithromycin	20	5/6 (83.3%)	2.16 ± 0.57
Clarithromycin:Pexiganan	20:10	6/6 (100%)	0
Clarithromycin:LLC	20	6/6 (100%)	0
Pexiganan:LLC	2	1/6 (17%)	5.12 ± 0.36
Pexiganan:LLC	5	2/6 (33.3%)	4.82 ± 0.16
Pexiganan:LLC	10	4/6 (67%)	3.61 ± 0.23

**Fig. 6:**
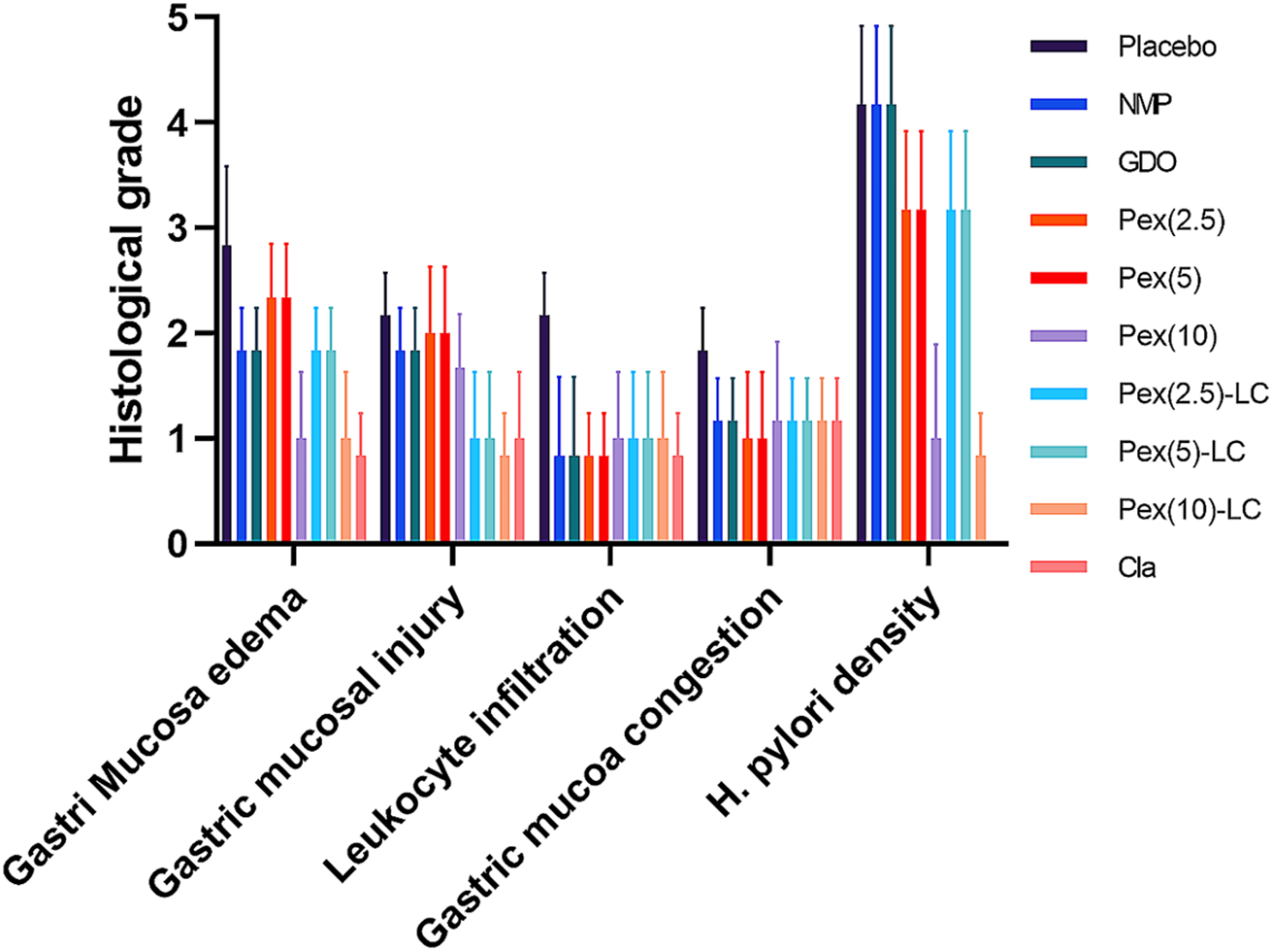
histopathological alteration grade in mice underwent intervention

Mechanical damage, such as gastric erosion, vascular congestion, infiltration of inflammatory cells, submocusa edema, and atrophy of gastric glands in the samples of mice without drug interference, was evident during the evaluation of histopathological changes in stomach samples. In the present study, clarithromycin and pexiganan treatments can result in the significant elimination of the observed conditions in mice. However, the intervention groups receiving pexiganan formulations without a vehicle exhibited some degree of inflammation, epithelial alterations, discontinuity of mucosa laminae, and atrophy of gastric glands, showing the administration of pexiganan without a suitable vehicle or formulation may lead to undesirable side effects and gastric tissue damage. In contrast, the use of clarithromycin may be a preferable and safer treatment option, showing the effective elimination of the observed conditions without any significant adverse effects on the gastric tissue. In this regard, clarithromycin has a more favorable risk-benefit profile compared to pexiganan formulations without a suitable vehicle or formulation, with some degree of tissue damage and inflammation due to pexiganan’s inability to have immunomodulatory effects. However, pexiganan alone did not have significant toxic effects on the stomachs of mice; the observed inflammation in the histopathological examination of the mouse stomach suggests that it could be due to the complete eradication of *Helicobacter pylori* infection from the mouse stomach by pexiganan. The appearance of inflammation in the histopathological evaluation of the stomachs of mice receiving pexiganan may be attributed to the presence of an unresolved *Helicobacter pylori* infection and the inflammatory response resulting from the presence of *Helicobacter pylori* in the mouse stomach. Furthermore, rapid clearance and degradation of carrier-free peptides may also disrupt the effectiveness of Pexiganan in suppressing and restoring the normal phenotype of gastric cells due to gastric acid. Histological evaluation after pexiganan-LLCs treatment revealed no epithelium alterations or incoherence of mucosa laminae. However, inflammatory cell infiltrations were observed in the submucosa tissue (Fig. 6).

## Discussion

In 1994, the WHO classified *H. pylori* as a class 1 carcinogen. Further, the risk of getting stomach cancer is higher in Asian, South American, and Eastern European populations due to the high incidence of *H. pylori* infection in these groups. Therefore, successful prevention and eradication of *H. pylori* infection can help decrease the incidence of gastric cancer (GC) (29–30). Antibiotic resistance as a problem has decreased the rate of *H. pylori* eradication and increased the prevalence of *H. pylori* infection [31]. The global *H. pylori* eradication rate has significantly decreased since 2010 [32]. Only 43% of the *H. pylori* strains studied in a multi-center investigation were completely sensitive across 18 European nations [33].

According to current research, the history of antibiotic usage and lifestyle factors play a significant role in influencing the antibiotic resistance pattern of *H. pylori* in different geographic areas. Accordingly, different populations may differently respond to various *H. pylori* treatment regimens (34–35). The AMPs, short peptides, can replace current antibacterial drugs. Vancomycin, colistin, and daptomycin are notable examples of AMPs, which are therapeutically used to treat infections and are also resistant to conventional drugs. These AMPs present promising alternatives in the battle against drug-resistant bacterial infections [36]. The current study aimed to assess the efficacy of pexiganan and its LLC formulation in treating *H. pylori* infection. The present study’s findings demonstrated the anti-*H. pylori* action of pexiganan. Furthermore, the eradication rate of *H. pylori* in mice is markedly increased by LLC-incorporated pexiganan. Moreover, this research clarified the significance of a medication delivery method tailored to the gastric mucosa in boosting pexiganan’s effectiveness during the *H. pylori* infection’s eradication.

The literature claims that pexiganan exhibits bactericidal properties by causing hole development to damage the bacterial cell membrane [37]. According to recent research, the cell membrane can be disrupted by pexiganan exposure in as little as five minutes [38]. For the first time, Iwahori demonstrated the impact of pexiganan on the proliferation of *H. pylori* ATCC43579 [39]. The research carried out by Ge and colleagues demonstrated that this peptide’s minimum inhibitory concentration (MIC) against *H. pylori* ATCC 43,504 was 2 µg/ml [8]. For three susceptible strains of *H. pylori*, Zhang et al. reported the minimum inhibitory concentration (MIC) of pexiganan and alginate-coated chitosan nanoparticles at 4 µg/ml [40].

Zhang demonstrated the concentration-dependent nature of pexiganan’s anti-*H. pylori* action, and even after 15 successive subcultures, no resistance was seen [40]. Our in vivo toxicity investigation showed that the maximal sub-lethal dosage of pexiganan for oral route in a mouse model was 10.7 mg/kg, which is in line with our findings. Significant toxicity from biochemical and hematological indicators, as well as normal histological examinations on the liver, stomach, and kidney, are not present at this dosage. However, recent research has shown that pexiganan is very hazardous to host cells [41–43], which is why topically applied pexiganan is most often used [44]. There’s a chance that the cell lines used in these studies are particularly vulnerable to this peptide (42–43). Furthermore, variables including the pH of the stomach and the turnover of the gastric mucosa might reduce the drug’s residence duration and contribute to its degradation, which raises the risk of treatment failure [45]. These issues can be greatly resolved and the chance of recovering from *H. pylori* infections increased by using a medication delivery method with stomach bioadhesive qualities [46]. Nikkam reported that in liposomes containing iron oxide, the inhibitory power of pexiganan does not significantly alter the encapsulated state [47]. Nevertheless, Fonseca demonstrated that the inhibitory activity of this formulation against *H. pylori* J99 was markedly increased by encasing pexiganan in chitosan nanoparticles [48].

Zhang et al. used alginate-coated chitosan nanoparticles to enhance the performance of pexiganan and revealed that nanoparticles do not significantly increase the elimination of *H. pylori* [40]. To the best of our knowledge, LLC-incorporated pexiganan formulations were used for the first time in this trial study to eradicate *H. pylori*. In comparison to other studies, the current study reveals that the use of prolonged-release and targeted distribution LLCs significantly enhances the eradication rate of *H. pylori* infection. The inconsistent results observed in different studies can be attributed to several factors, including variations in *H. pylori* strains, the amino acid sequence of pexiganan, different methods of medication delivery, and varying dosages. Under PLM, the LLC-pexiganan structures exhibited a hexagonal liquid crystal configuration. The LLC-H2 phase is particularly suitable for slow-release formulations [49]. However, the clearance rate of *H. pylori* infection in mouse models that received clarithromycin was higher than that with Pex-LLCs formulations. Clarithromycin has more incomparable efficacy than antimicrobial peptides for the cure of *H. pylori* infection, particularly in mouse models [10].

Moreover, lipid-liquid crystal structures protect the hydrophobic compounds inside their nano-channels and carry significant amounts of drugs of different sizes due to the increased surface ratio [50]. The initial burst release phenomenon was one of the most important reasons for the difference in the eradication rate of *H. pylori* in the groups receiving pexiganan, so that the non-vehicle groups could not eradicate *H. pylori* due to the short residence of pexiganan within the gastric mucosa. Similarly, the mucoadhesive nature of LLC-pexiganan formulations may have potentially increased the bioavailability of pexiganan and extended its half-life in the stomachs of mice. However, it is important to highlight that the present investigation did not assess the bioavailability and half-life of pexiganan. Further studies are needed to evaluate these pharmacokinetic parameters and to comprehensively understand the impact of LLC-pexiganan formulations on the delivery and stability of the peptide in the stomach. In the proposed formulation in the current study, PC accounted for about half the weight of LLC. Similar studies indicate that PC in high percentage plays a key role in phase inversion towards hexagonal and anisotropic and thus optimization of drug release [51].

The results of the present study revealed a reduction in gastric inflammation in mice receiving LLC-pexiganan compared to placebo, and there is a minimum epithelial cell alternation and infiltration of inflammatory cells in the gastric submucosa compared to placebo. Recent studies have indicated that the LLC formulations are generally safe and their side effects are very rare (52–53). The obtained results suggested liquid crystal attenuates gastric histological damage, exemplifying the clinical safety of this formulation in animal models as a representative alternative option for the development of novel antimicrobial agents. In addition, the slow release of pexiganan in this formulation can lead to more time for the complete restoration of gastric inflammation. Therefore, clarithromycin is superior than pexignan and its lipid liquid crystal in the eradication of *H. pylori* infection. There are several restrictions on this study. For instance, one of the most significant problems is the absence of immunohistochemistry staining on stomach tissue samples. Additionally, the current investigation did not examine the efficacy of our therapeutic lipid-liquid crystal formulations on drug-resistant strains of *H. pylori*. Apart from the minimum number of mice assigned to each group, we did not measure the development of resistance to clarithromycin and pexiganan during continuous sub-cultures, and there was a lack of bioavailability and half-life of pexiganan measures (e.g., both loaded in LLCs and without vehicles) in the stomach of mice. Thus, further research is still required to further solidify.

## Conclusion

In vitro and in vivo investigations show that GDO-based formulations produce prolonged release of pexiganan. The LLC-pexiganan monotherapy does not have the potential to successfully eliminate *H. pylori* infection. However, the recent data demonstrate that the peptide can improve the effectiveness of conventional *H. pylori* therapy regimens. Furthermore, the findings highlight clarithromycin’s greater efficacy in totally eradicating *H. pylori* infection as compared to pexiganan. However, further research is needed to assess the impact of pexiganan, particularly in animal model studies.

## Data Availability

The datasets generated and/or analysed during the current study are available in the NCBI database repository, (Accession number: MK515150).

## Abbreviations

*H. pylori*: *Helicobacter pylori*
LLCs: Lipid-liquid crystals
MALT: Mucosa-associated lymphoid tissue
MDR: Multidrug-resistant
WHO: World Health Organization
AMPs: Antimicrobial peptides
LCNs: Liquid crystal nanoparticles
GDO: Glycerol dioleate
GI: Gastrointestinal tract
PLM: Polarized light microscope
GC: Gastric cancer
